# Thrombolytic Dilemma: A Case Report of Early Puerperium Ischemic Stroke Treated With Intravenous Thrombolysis

**DOI:** 10.7759/cureus.33204

**Published:** 2023-01-01

**Authors:** André Santos, Maria Lima Costa, José Eduardo Sousa, Ilídia Carmezim, Ana Gomes

**Affiliations:** 1 Internal Medicine, Centro Hospitalar Tondela Viseu, Viseu, PRT; 2 Internal Medicine, Centro Hospitalar Tondela-Viseu, Viseu, PRT; 3 Physical Medicine and Rehabilitation, Centro de Medicina de Reabilitação da Região Centro - Rovisco Pais, Figueira da Foz, PRT; 4 Stroke Unit, Centro Hospitalar Tondela-Viseu, Viseu, PRT; 5 Stroke Unit, Centro Hospitalar Tondela-Viseu, viseu, PRT

**Keywords:** postpartum complication, early puerperium thrombolysis, tissue plasminogen activator, thrombolytic therapy, ischemic stroke

## Abstract

A 25-year-old woman was admitted to the obstetrics ward when presented with a sudden onset of expressive aphasia and minor right facial palsy 48 hours after forceps-assisted delivery. The intrahospital emergency team was immediately mobilized. The patient had a blood pressure (BP) of 119/79 mmHg, heart rate of 114 bpm, O_2_ saturation of 97%, and blood glucose level of 136 mg/dL. Trauma and toxic exposure were ruled out. A rapid EKG was performed with no significant changes. Assuming an acute stroke, the patient immediately underwent brain CT (approximately 15 minutes after the beginning of the symptoms), which revealed no signs of hemorrhage, an ischemic area, or masses. Brain CT angiography was then performed; however, no major brain artery obstruction was found. With brain hemorrhage ruled out and persistent neurologic deficits, the case was discussed between the emergency team doctor and the patient’s obstetrician, and intravenous thrombolysis (IVT) with recombinant tissue plasminogen activator (rt-PA) was started approximately 45 minutes after the onset of symptoms. After treatment completion, the patient had a complete resolution of the neurological deficits. The patient remained under strict observation at the acute stroke unit (ASU), and no secondary brain hemorrhage or post-partum-related complications were noted.

## Introduction

Stroke can be categorized as intracerebral hemorrhage, subarachnoid hemorrhage, or ischemic stroke. Although the clinical course of symptoms may be suggestive of the type of stroke, neuroimaging is required to reliably differentiate between intracerebral hemorrhage and ischemic stroke. The most common presenting symptoms of ischemic stroke are difficulty with speech, facial palsy, and weakness in one half of the body [1]. Pregnancy and the puerperium (until six weeks after delivery) are known to increase the risk of stroke, especially during late pregnancy and during the puerperium [2]. During this period of pregnancy, women have the highest risk of hypertensive disorders and gestational hypercoagulability. This hypercoagulable state may be attributed to decreased protein S and antithrombin III activity and increased von Willebrand factor, fibrinogen, factor VIII, plasminogen activator inhibitors, protein C resistance, and platelet aggregation [3]. Venous stasis, cerebral aneurysm, endothelial injury, postpartum angiopathy, and cerebral venous thrombosis are other known possible etiologies of pregnancy-associated stroke [4]. The incidence of stroke during pregnancy and the puerperium is widely variable, with a reported range between 5 and 67 per 100,000 pregnancies [C]. Thromboembolic events represent one of the major causes of maternal death during the postpartum period. Stroke is rare in pregnancy and puerperium but still accounts for 12% of maternal death causes [4]. Postpartum thrombolytic therapy with rt-PA is controversial because of the fear that the treatment may lead to massive bleeding. Nevertheless, a few case reports have shown the beneficial effect of thrombolysis on acute ischemic stroke (AIS) in pregnancy [5].

## Case presentation

**Figure 1:**
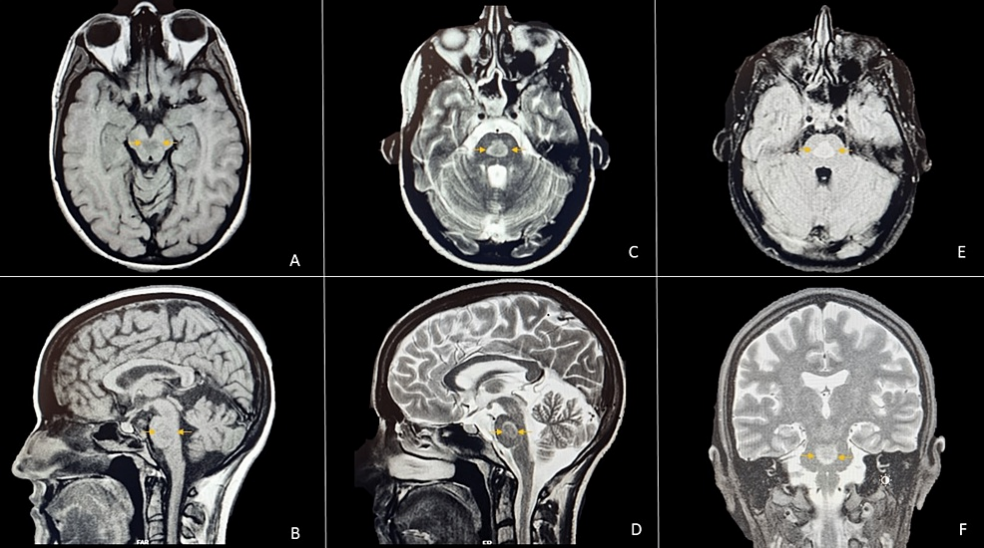
Brain magnetic resonance imaging MRI revealing a median pontine rounded lesion (yellow arrows) without mass effects, with slight T1 signal attenuation (A - axial T1; B - sagital T1), T2 hypersignal (C - axial T2; D - sagital T2; F - coronal T2) and hyperintensity on fluid-attenuated inversion recovery (FLAIR) imaging (E - axial FLAIR) suggestive of late subacute ischemic etiology.

**Figure 2:**
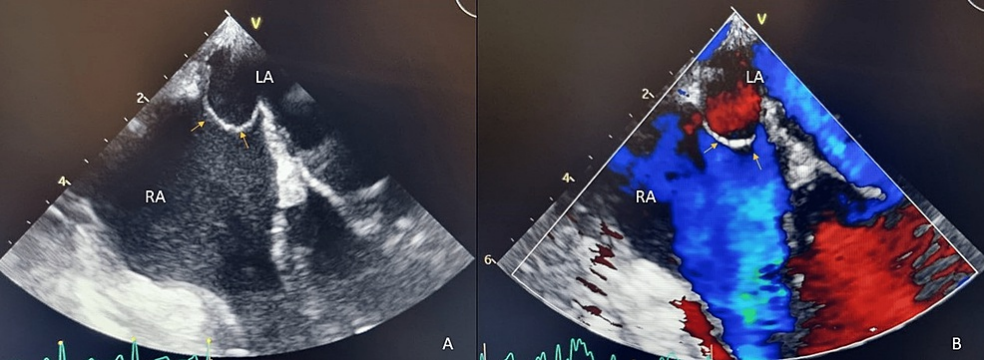
Transoesophageal echocardiography 2D transoesophageal echocardiography image (A) of a patent foramen ovale (yellow arrows), with evident shunting on the color flow doppler (B). RA: right atrium; LA: left atrium.

Here, we describe a case of a 25-year-old postpartum woman with a known history of subclinical hyperthyroidism admitted to the obstetrics ward. The patient presented with sudden onset of speech difficulty 48 hours after forceps-assisted delivery. The nursing team promptly informed the intrahospital emergency team. At the first observation, the patient presented with expressive aphasia and minor right upper motor neuron (UMN) facial palsy. The patient’s BP was 119/79 mmHg with a heart rate of 114 bpm, an O2 saturation of 97%, and a blood glucose level of 136 mg/dL. A bedside EKG showed a sinus rhythm with no relevant findings. Trauma and toxic exposure were ruled out. The patient was only given 40 mg of enoxaparin daily for thromboembolism prophylaxis. The last administered dose was around 13 hours before the incident. Assuming an acute stroke, the National Institutes of Health Stroke Scale (NIHSS) was used, and the patient had a score of 3 points (aphasia - 2 points; minor facial paralysis - 1 point). The patient immediately underwent a brain CT (15 minutes after the onset of symptoms), which showed a normal result. With brain hemorrhage ruled out and persistent neurologic deficits, the case was discussed between the emergency team doctor and the patient’s obstetrician, and intravenous thrombolysis (IVT) with alteplase (rT-PA) was started 45 minutes after the beginning of the symptoms. After treatment completion, the patient had a complete resolution of the initial symptoms. Brain CT angiography was performed, which showed no signs of significant strictures or thrombi on the main neck and intracerebral arteries. The patient remained under close observation at the Acute Stroke Unit (ASU). A post-IVT brain CT scan at 24 hours ruled out signs of procedure-related complications or signs of an established infarction. A few days later, the patient underwent MRI (Figure 1), which revealed a median pontine rounded lesion without mass effects, with slight T1 signal attenuation and hypersignal on long TR sequences, and with slight restriction of proton diffusion, suggesting a possible late subacute ischemic etiology. No post-partum-related complications were noted until patient discharge. Ambulatory transthoracic echocardiography and neck vessel doppler ultrasonography did not detect any abnormalities. Thrombophilia screening performed after two months also showed a negative result. Transoesophageal echocardiography (Figure 2) revealed a patent foramen ovale with major thrombotic risk. The patient was prescribed a direct-acting oral anticoagulant (DOAC), and there were no notable events after five months.

## Discussion

The risk of AIS is known to increase in the postpartum period. Although the incidence of stroke during pregnancy and the puerperium has risen in recent years (50% in the last 20 years), the reported incidence of stroke varies widely [6]. Several pre-existing medical conditions are related to the increased risk of stroke, which includes diabetes mellitus, hypertension, heart disease, sickle cell disease, antiphospholipid syndrome, thrombophilia, and migraine [7]. Several pregnancy-related complications such as postpartum hemorrhage, amniotic fluid embolism, pre-eclampsia, eclampsia, and peripartum cardiomyopathy have also been associated with an increased stroke incidence. For non-pregnant patients with AIS, early endovascular clot retrieval and thrombolytic therapy are the recommended acute therapies to improve long-term clinical outcomes. However, the efficacy of these therapies has not been confirmed in randomized trials involving pregnant women. Moreover, these therapies are often withheld for most women given concerns for life-threatening maternal and placental hemorrhage, including the risk of fetal demise [8]. Thus far, no relevant studies have established the safety of thrombolysis during the early postpartum period. Thrombolysis during pregnancy and the puerperium has usually been excluded from AIS trials with intravenous rt-PA or intra-arterial thrombolytic therapy. A review published by Akazawa and Nishida presented the data from 13 cases of systemic thrombolytic therapy during the early postpartum period (48 hours postpartum). They reported that blood transfusion was required in 12 of the cases (92%). Subsequent laparotomy to control bleeding was required in five cases (38%), with hysterectomy in three of them and hematoma removal in the other two cases. Interestingly, all of these cases involved cesarean delivery. In seven cases (54%), large-volume transfusions were required. No laparotomy was reported in cases of transvaginal delivery. The occurrence of severe bleeding was higher in cesarean section deliveries compared with vaginal deliveries. Therefore, using rt-PA along with cesarean delivery might be worth avoiding. However, the limited data in the literature make it difficult to assess the final outcome and safety of this treatment [9]. The MBRRACE-UK Perinatal Mortality Surveillance Report indicated that pregnancy, cesarean section delivery, and the immediate postpartum state are not absolute contraindications to thrombolysis (intra-arterial or intravenous) [10]. Some experts suggest that postpartum women who develop disabling ischemic stroke occurring at least 10 days after delivery, who otherwise meet eligibility criteria, can be treated with rt-PA after appropriate benefit/risk profile assessment on an individual basis; if endovascular thrombectomy is available and indicated, it should be the preferred method [11].

In the presented case, it should be mentioned that our hospital center does not have easy access to endovascular thrombectomy and no major vessel obstruction was detected on the brain CT angiography. Therefore, the only available treatment option for this patient was IVT with rt-PA. In this setting, it is also worth mentioning that although the patient was taking enoxaparin, guidelines emphasize the use of low molecular weight heparin (LMWH) as an absolute contraindication to IVT with rt-PA but only when given in therapeutic doses (1 mg/kg twice daily). There is no strong evidence regarding DVT prophylaxis doses although it should be carefully evaluated. We have extensive experience dealing with ischemic stroke but only a few pregnancy-related cases. Nevertheless, our team is aware that the early postpartum period is critical, in which the need for thrombolysis may arise. There was no record of early puerperium thrombolysis in our center. In this setting, a careful discussion was conducted between the obstetric team on duty, the stroke expert, and the patient. Although the NIHSS score was relatively low (NIHSS=3), expression aphasia is a very limiting deficit for a 25-year-old person; thus, treatment was initiated. It should be noted that the decision was made considering that this was not a cesarean partum; otherwise, an increased risk of maternal death due to hemorrhagic complications would have prevented this decision. Although the available data on stroke management does not provide a specific recommendation for these cases, the available information derived from postpartum pulmonary thromboembolism cases seems to be consistent with the given treatment [11].

## Conclusions

The presented patient was young and had a high thrombotic risk due to a patent foramen ovale, which was unknown. Although the risk of early postpartum ischemic stroke is higher in cesarean section deliveries, it can also occur in normal deliveries in patients with no other thrombotic risk factors. Due to the lack of safety research, stroke management for affected patients is always a stressful experience and poses tremendous challenges for clinicians. It should be kept in mind that prevention is always the best approach. The identification and monitoring of all high-risk women in the postpartum period for thrombotic complications should be carried out. After careful assessment of the risk/benefit ratio, prophylactic anticoagulant therapy should be considered. At this point, more studies are required to determine the optimal duration of prophylaxis. Regardless of the constraints, fortunately, this patient’s outcome was excellent.
